# Updated Meta-Analysis of Randomized Controlled Trials Comparing External Fixation to Intramedullary Nailing in the Treatment of Open Tibial Fractures

**DOI:** 10.3390/medicina59071301

**Published:** 2023-07-14

**Authors:** Danilo Jeremić, Nina Rajovic, Boris Gluscevic, Branislav Krivokapic, Stanislav Rajkovic, Nikola Bogosavljevic, Kristina Davidovic, Slavko Tomic

**Affiliations:** 1 Institute for Orthopedic Surgery “Banjica”, 11000 Belgrade, Serbia; glborismmm@gmail.com (B.G.); branislav.krivokapic@iohbb.edu.rs (B.K.); stanbgd@hotmail.com (S.R.); boga19@gmail.com (N.B.); tomicslavko1956@gmail.com (S.T.); 2 Faculty of Medicine, University of Belgrade, 11000 Belgrade, Serbia; dr.kristina.davidovic@gmail.com; 3 Institute for Medical Statistics and Informatics, Faculty of Medicine, University of Belgrade, 11000 Belgrade, Serbia; nina.rajovic@med.bg.ac.rs; 4 Department of Radiology, Clinical Center of Serbia, 11000 Belgrade, Serbia

**Keywords:** meta-analysis, open tibial fractures, external fixator, intramedullary nailing

## Abstract

*Background:* The purpose of this study was to collect all available randomized controlled trials (RCT) on the treatment of open tibial fractures with an external fixator (EF) and intramedullary nailing (IMN) for meta-analysis to provide reliable evidence-based data for clinical decision-making. *Material and methods:* The systematic review was undertaken in accordance with Preferred Reporting Items for Systematic Reviews and Meta-Analyses (PRISMA) and AMSTAR (Assessing the Methodological Quality of Systematic Review). An electronic search of PubMed, Cochrane Library, and Web of Science was performed until 1 March 2023 to identify RCTs which compared either IMN or EF to fix the open tibial fracture. Outcome measures were: postoperative superficial and deep infection, time to union, delayed union, malunion, nonunion and hardware failure. In addition, pain and health-related quality of life were evaluated after 3 and 12 months of follow-up. *Results:* Sixteen publications comprising 1011 patients were included in the meta-analysis. The pooled results suggested that the IMN technique had a lower postoperative superficial infection and malunion rate (RR = 3.56, 95%CI = 2.56–4.95 and RR = 1.96, 95%CI = 1.12–3.44, respectively), but higher hardware failure occurrence in contrast to EF (RR = 0.30; 95%CI = 0.13–0.69). No significant differences were found in the union time, delayed union or nonunion rate, and postoperative deep infection rate between the treatments. Lower levels of pain were found in the EF group (RR = 0.05, 95%CI = 0.02–0.17, *p* < 0.001). A difference in quality of life favoring IMN after 3 months was found (RR = −0.04, 95%CI = −0.05–0.03, *p* < 0.001), however, no statistical difference was found after 12 months (RR = 0.03, 95%CI = −0.05–0.11, *p* = 0.44). *Conclusions:* Meta-analysis presented reduced incidence rates of superficial infection, malunion, and health-related quality of life 3 months after treatment in IMN. However, EF led to a significant reduction in pain and incidence rate of hardware failure. Postoperative deep infection, delayed union, nonunion and health-related quality of life 12 months following therapy were similar between groups. More high-quality RCTs should be conducted to provide reliable evidence-based data for clinical decision-making.

## 1. Introduction

The most common type of open fracture of the long bones in the extremities is the open tibial fracture, which is frequently observed in traffic accidents [1,2]. For patients suffering from this type of fracture, emergency wound debridement, exploration of vascular and nerve damage, early soft tissue coverage, and fracture stabilization are agreed-upon treatments [3,4,5]. Two common surgical methods used to treat this fracture are external fixators (EF) and intramedullary nailing (IMN). However, both methods have their own advantages and disadvantages, making it controversial which one is better [6].

In the past, EF was widely used due to its rapid operation, lack of surgical incision, and no negative effect on blood supply to the fracture site [7]. However, postoperative patients with EF often suffer from complications such as pin-track infections, fracture malunion, reduction loss, and joint contracture [8,9]. Additionally, the long-term use of EF can be inconvenient for nurses and patients. Nowadays, IMN is widely used because of its advantages of central fixation, early weight-bearing, minimal invasiveness, and convenient postoperative care [10,11]. However, there are still risks of hardware failure and infection diffusion through the medullary cavity [12].

Several meta-analyses on the treatment of open tibial fractures with IMN and EF were conducted, but there have been limitations. Some did not compare fracture healing time [13], others did not conduct heterogeneity analysis [14], and some included retrospective studies and case reports [15], which impacted the level of evidence. Aiming to collect the best available evidence, Liu et al. published meta-analysis based on randomized clinical trials (RCTs) comparing the treatment of open tibial fractures with EF and IMN, and recommended IMN as a preferred method of fracture fixation for patients with open tibial fractures [16]. However, none of these meta-analyses assessed the level of pain and quality of life of patients undergoing EF or IMN, which may influence decisions about treatment modalities. In addition, a new RCT on functional and radiological outcomes of primary ring fixator versus antibiotic nails in open tibial diaphyseal fractures has been reported recently [17]. Therefore, the purpose of this study was to collect all available RCTs on the treatment of open tibial fractures with EF and IMN for meta-analysis to provide reliable evidence-based data for clinical decision-making.

## 2. Materials and Methods

The systematic review was undertaken in accordance with Preferred Reporting Items for Systematic Reviews and Meta-Analyses (PRISMA) [18,19] and AMSTAR (A Measurement Tool to Assess Systematic Reviews) [20] (Supplementary Material Tables S1 and S2). Review methods were established prior to the conduct of the review and there were no significant deviations from the protocol.

### 2.1. Study Selection

Screening for inclusion of publications in the systematic review was performed in two phases, with the discussion or consensus of two reviewers at each stage and with the inclusion of a third reviewer to resolve all possible discrepancies. Eligible studies were published RCT trials which compared the use of either IMN or EF to fix the tibial fracture. Studies were excluded if: (1) did not make a comparison between IMN and EF; (2) had other populations (animal, femur etc.); (3) assessed other techniques in fixing tibial fracture; (4) did not have the outcomes of interest; (5) were abstracts; (6) were not original articles; (7) were not confined to the English language.

### 2.2. Search Strategy

The search strategy was developed by two reviewers, one with a background in orthopedics and one with experience in developing search strategy. An electronic search of databases such as PubMed, Cochrane Library, and Web of Science until 1 March 2023 was conducted to identify published studies containing the following keywords: “fracture external fixation” and “tibial intramedullary nailing”.

### 2.3. Data Abstraction and Quality Assessment

The following data were abstracted independently by two reviewers: title of the study, author(s), year of publication, country where research was performed, sample size, gender and age of patients included, duration of follow-up, intervention type, and types of fracture. Data of the following outcomes of interest were abstracted: presence of superficial and deep infections, union time, delayed union, malunion, nonunion, and hardware failure. Health-related quality of life data measured using the EQ-5D and data on the presence of pain were additionally obtained from the related articles. If data were unclear or missing, the authors of relevant articles were contacted.

### 2.4. Risk of Bias

The risk of bias within each study and the overall quality of the gathered evidence was assessed independently by two reviewers using a quality assessment The Risk of Bias 2 (RoB 2) tool of “Cochrane Collaboration’s tool to assess the risk of bias in randomized trials“. The domains included in RoB 2: bias arising from the randomization process, bias due to deviations from intended interventions, bias due to missing outcome data, bias in the measurement of the outcome, and bias in the selection of the reported result covered all types of bias that are currently understood to affect the results of RCTs [21]. Publication bias was assessed by funnel plots (Supplementary Material Figure S1). The sources of funding for individual studies included in the review were not reported by the study authors.

### 2.5. Data Analysis

Analyses of the included studies were performed using Review Manager Version 5.4 (Cochrane, 2021). Continuous outcomes, such as time to union and health-related quality of life were expressed with mean difference (MD) and 95% confidence interval (CI). The mean difference for time to union was calculated as MD = µIMN − µEF, whereas the mean difference for health-related quality of life was calculated as MD = µEF − µIMN. If continuous data were presented with mean and ranges, standard deviations were estimated as (max-min)/6. Dichotomous variables, such as postoperative superficial and deep infection, delayed union, malunion, nonunion, hardware failure, and pain were expressed by risk ratio (RR) and 95% CI. The risk ratio was calculated as the ratio of the risk of postoperative superficial and deep infection, delayed union, malunion, and nonunion in the EF group in contrast to the IMN group. The risk ratio was calculated as the ratio of the risk of hardware failure and pain in the IMN group in contrast to the EF group. Chi-square Q and I^2^ statistics were used to assess heterogeneity. Based on the Cochrane Handbook [22], I2 categorization of heterogeneity states that I^2^ < 30% corresponds to low, I^2^ = 30–60% corresponds to moderate, and I^2^ > 60 corresponds to high heterogeneity of the included studies. Fixed-effect analysis and random-effect analysis were used for data with low and high heterogeneity, respectively. For each analysis, a separate forest plot was constructed, showing the RR (box), 95% CI (lines), and weight (size of box) for each study. The overall effect size was represented by a diamond. A *p*-value of <0.05 was considered to be statistically significant for all analyses.

## 3. Results

### 3.1. Search Results

**Figure 1:**
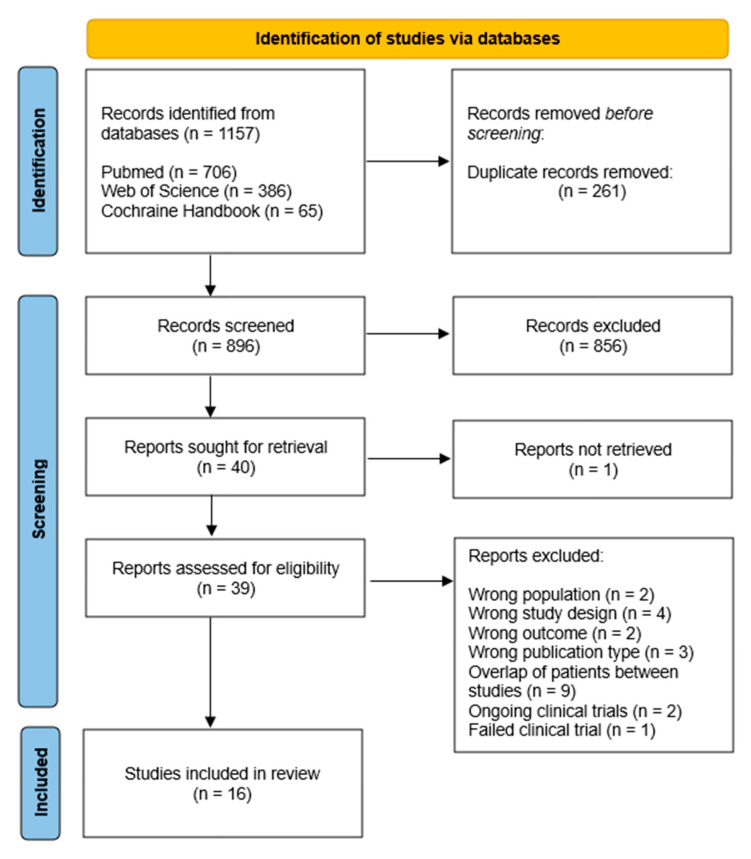
Flowchart of the study selection process.

A total of 1157 potentially eligible articles were extracted from three electronic databases. After duplicates were removed, 896 titles and abstracts were screened for relevance. Eventually, 856 studies did not meet the eligibility criteria and a total of 40 articles were sought for retrieval. Due to one article not being retrieved, 39 articles were assessed for eligibility. After screening the full text, two studies were excluded because they had a wrong population, four had the wrong study design, two had the wrong outcome, three were the wrong publication type, nine represented follow-up studies, two were ongoing clinical trials, and one clinical trial failed. The study selection process using the PRISMA flow diagram is shown in Figure 1.

### 3.2. Characteristics of Eligible Studies

**Figure 2:**
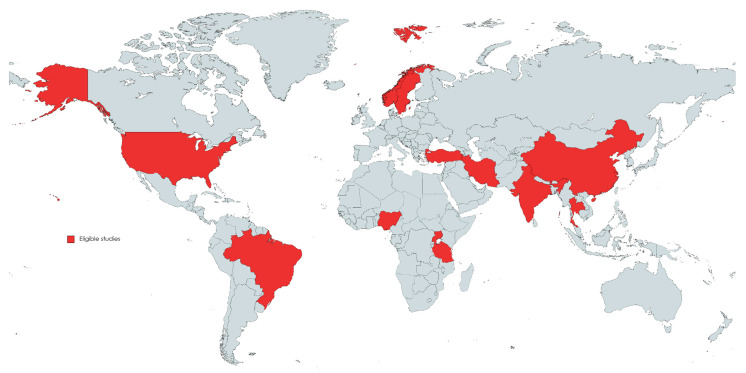
Geographical overview of the eligible studies included in the meta-analyses.

Characteristics of all 16 publications included in the meta-analysis are presented in detail in Table 1. The studies were published between 1994 and 2022, with a minimum sample size of 29 [23] and a maximum of 221 [24]. Five eligible studies were conducted from Asian countries, three from African countries, four from European countries, three from the USA, and one eligible study was conducted from South America (Figure 2). The average age varied from 25 years to 46 years, and studies comprised a total of 1011 patients (811 male and 200 female). The average duration of follow-up ranged from 4.5 to 46.5 months, and in eligible studies fracture types ranged from I to IIIb according to the Gustilo–Anderson classification.

### 3.3. Quality Assessment of the Eligible Studies

**Figure 3:**
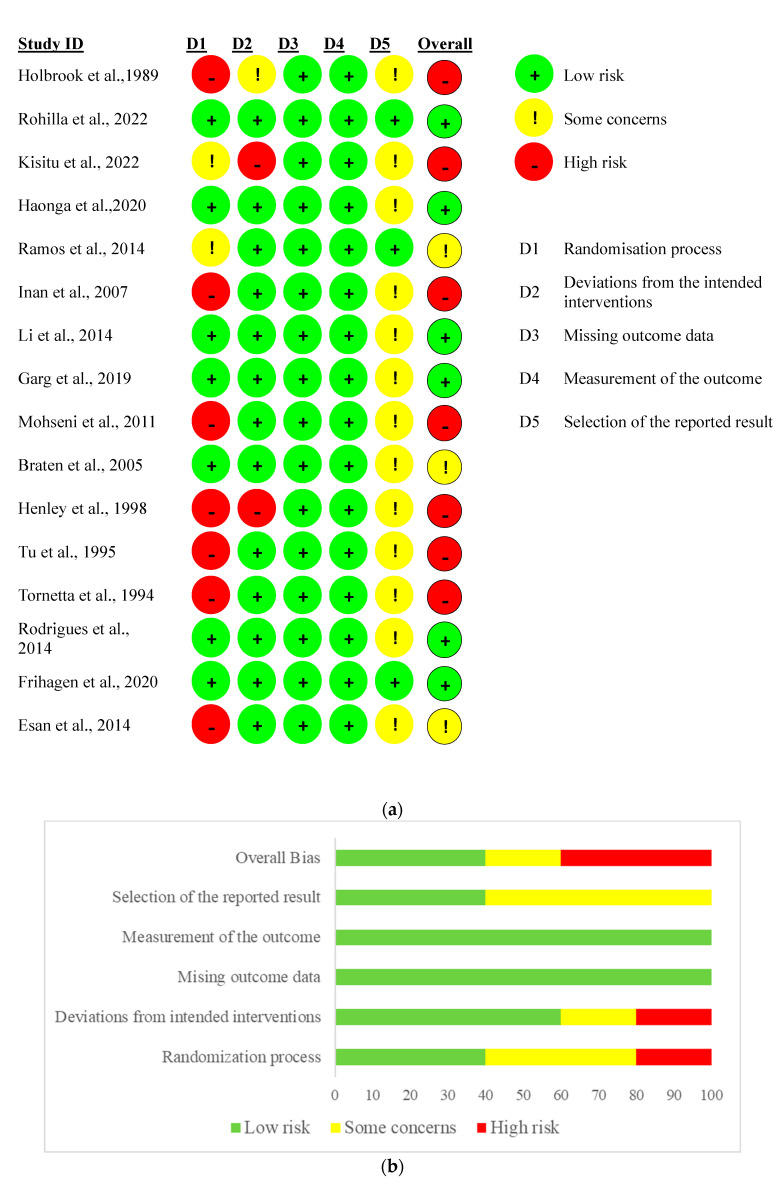
(**a**) Risk of bias according to domains [8,23,24,25,26,27,28,29,30,31,32,33,34,35,36,37]. (**b**) Overall risk of bias.

Sixteen studies which reported the randomization method [8,23,24,25,26,27,28,29,30,31,32,33,34,35,36,37] were assessed for the risk of bias according to the Cochrane Handbook. Six studies reported that the method of randomization in their study was based on even or odd medical record number [8,23,25,31,33,34]. In studies conducted by Kisitu et al. [27], Ramos et al. [28], Li [29], Braten et al. [32], and Rodrigues [35], sealed opaque envelopes were used for randomization; Li et al. [29] stated that the randomization was performed by computer allocation and that sequentially numbered opaque envelopes were assigned to included patients prospectively. However, Kisitu et al. [27] reported in their study that computer randomization was not logistically viable, therefore opaque envelopes were sorted in random sequence. A centralized web-based electronic randomization tool was the randomization method used in the study by Rohilla et al. [26], Haonga et al. [24], and Frihagen et al. [36]. Garg et al. [30] reported that the randomization chit box was used, whereas Esan et al. [37] randomized patients with simple random sampling using a balloting process. In the study conducted by Kisitu et al. [27], it was stated that patients and the treating staff were not blinded to their study group allocation. All studies showed a low risk of bias due to missing outcome data and the measurement of the outcome. Detailed information about the quality assessment of the eligible studies is shown in Figure 3a,b.

## 4. Results of Meta-Analysis

### 4.1. Postoperative Superficial Infection

**Figure 4:**
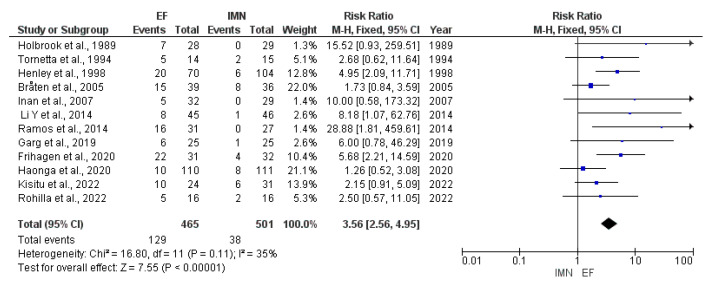
Forest plot presenting the comparison of postoperative superficial infections occurrence between EF and IMN; Blue squares—the individual study effect; Diamond—pooled effect [8,23,24,25,26,27,28,29,31,32,34,36].

Twelve studies [8,23,24,25,26,27,28,29,30,32,33,36] with a total of 966 cases (EF = 465, IMN = 501) reported the presence of postoperative superficial infection. The fixed-effects model was used due to low heterogeneity among studies (I^2^ = 35%). The presence of postoperative superficial infection was significantly higher in the EF group compared to the IMN group (RR = 3.56, 95%CI = 2.56–4.95, *p* < 0.001) (Figure 4).

### 4.2. Postoperative Deep Infection

**Figure 5:**
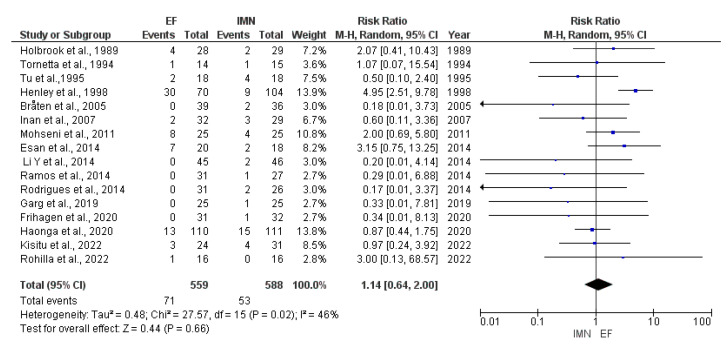
Forest plot presenting the comparison of postoperative deep infections occurrence between EF and IMN; Blue squares—the individual study effect; Diamond—pooled effect [8,23,24,25,26,27,28,29,30,31,32,33,34,35,36,37].

Data on postoperative deep infection were provided by all sixteen studies [8,23,24,25,26,27,28,29,30,31,32,33,34,35,36,37] included in the meta-analysis. Due to moderate heterogeneity (I^2^ = 46%) among included studies, random-effect analysis was performed, and the results showed that no statistical difference was found in the presence of postoperative deep infections between EF and IMN groups (RR = 1.14, 95%CI = 0.64–2.00, *p* = 0.66) (Figure 5).

### 4.3. Time to Union

**Figure 6:**
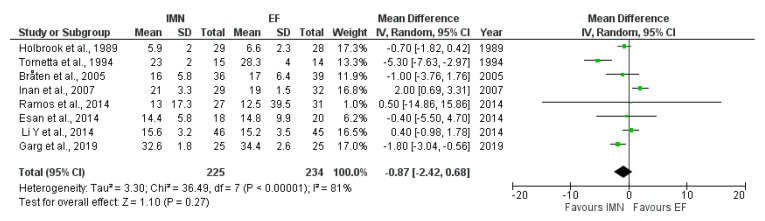
Forest plot presenting the mean difference in time to union between EF and IMN; Green squares—the individual study effect; Diamond—pooled effect [8,23,27,28,29,31,34,37].

Eight studies [8,23,25,28,29,30,32,37] comprising a total of 459 cases (EF = 234, IMN = 225) provided data about union time. The random-effects model was adopted due to high heterogeneity among studies (I^2^ = 81%). The results of meta-analysis showed no statistical difference in time to union between IMN and EF groups (MD = −0.87, 95%CI = −2.42–0.68), *p* = 0.27) (Figure 6).

### 4.4. Delayed union

**Figure 7:**
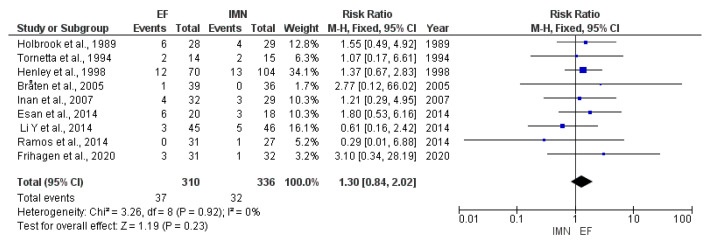
Forest plot presenting the comparison of delayed union occurrence between EF and IMN; Blue squares—the individual study effect; Diamond—pooled effect [8,23,27,28,31,32,34,36,37].

Data on delayed union were provided by nine studies [8,23,25,28,29,32,33,36,37] included in the meta-analysis. Due to no heterogeneity (I^2^ = 0%) among included studies, fixed-effect analysis was performed, and the results showed that no statistical difference was found in the presence of delayed union between EF and IMN groups (RR = 1.30, 95%CI = 0.84–2.02, *p* = 0.23) (Figure 7).

### 4.5. Malunion

**Figure 8:**
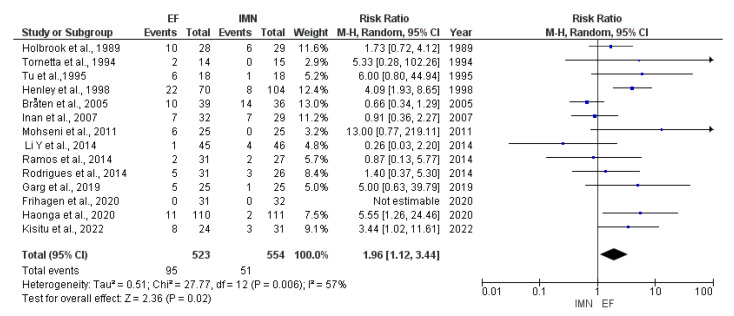
Forest plot presenting the comparison of malunion occurrence between EF and IMN; Blue squares—the individual study effect; Diamond—pooled effect [8,23,25,26,27,28,29,30,31,32,33,34,35,36].

Fourteen studies [8,23,24,25,27,28,29,30,31,32,33,34,35,36] with a total of 1077 cases (EF = 523, IMN = 554) reported the presence of malunion. The random-effects model was used due to high heterogeneity among studies (I^2^ = 57%). The results of the meta-analysis showed significant difference in malunion occurrence between EF and IMN group, favoring IMN (RR = 1.96, 95%CI = 1.12–3.44, *p* = 0.02) (Figure 8).

### 4.6. Nonunion

**Figure 9:**
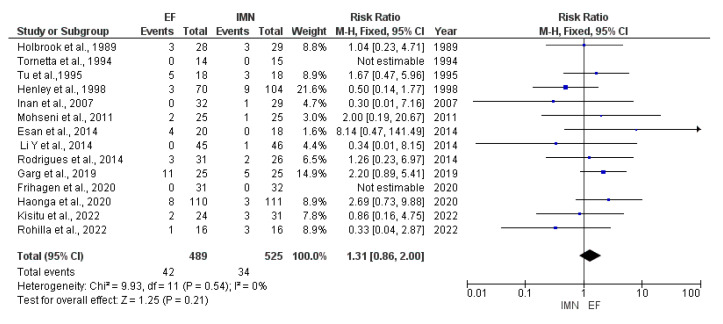
Forest plot presenting the comparison of nonunion occurrence between EF and IMN; Blue squares—the individual study effect; Diamond—pooled effect [8,23,24,25,26,28,29,30,32,33,34,35,36,37].

Data on nonunion were provided by fourteen studies [8,23,24,25,26,27,29,30,31,33,34,35,36,37] included in the meta-analysis. Due to no heterogeneity (I^2^ = 0%) among included studies, fixed-effect analysis was performed, and the results showed that no statistical difference was found in the presence of nonunion between EF and IMN groups (RR = 1.31, 95%CI = 0.86—2.00, *p* = 0.21) (Figure 9).

### 4.7. Hardware Failure

**Figure 10:**
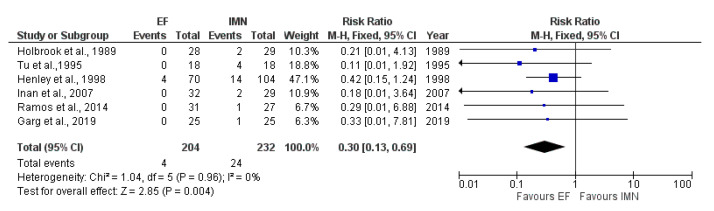
Forest plot presenting the comparison of hardware failure occurrence between EF and IMN; Blue squares—the individual study effect; Diamond—pooled effect [8,23,27,29,32,33].

Six studies [8,25,28,30,33,34] with a total of 436 cases (EF = 204, IMN = 232) reported the presence of hardware failure. The fixed-effects model was adopted due to no heterogeneity among studies (I^2^ = 0%). The results of the meta-analysis showed significant difference in hardware failure between EF and IMN group, favoring EF (RR = 0.30, 95%CI = 0.13–0.69, *p* = 0.004) (Figure 10).

### 4.8. Pain

**Figure 11:**
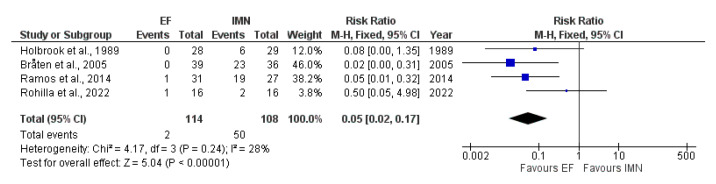
Forest plot presenting the comparison of pain occurrence between EF and IMN; Blue squares—the individual study effect; Diamond—pooled effect [23,24,27,31].

Data on pain were provided by four studies [25,26,28,32] included in the meta-analysis. Due to low heterogeneity (I^2^ = 28%) among included studies, fixed-effect analysis was performed, and the results showed that statistical difference was found in the presence of pain between EF and IMN groups, favoring EF (RR = 0.05, 95%CI = 0.02–0.17, *p* < 0.001) (Figure 11).

### 4.9. Health-Related Quality of Life Measured after 3 Months

**Figure 12:**
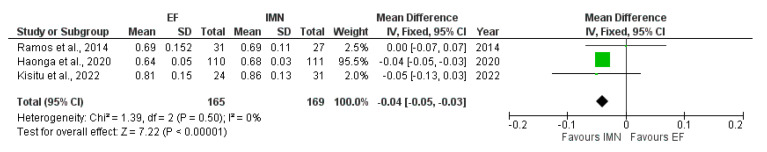
Forest plot presenting the comparison of Health-related quality of life measured after 3 months between EF and IMN; Green squares—the individual study effect; Diamond—pooled effect [25,26,27].

Three studies [24,27,28] with a total of 334 cases (EF = 165, IMN = 169) provided data about the quality of life related to health. The fixed-effects model was adopted due to no heterogeneity among studies (I^2^ = 0%). The results of the meta-analysis showed a significant difference in quality of life between EF and IMN groups after 3 months of procedure, favoring IMN (RR = −0.04, 95%CI = −0.05–0.03, *p* < 0.001) (Figure 12).

### 4.10. Health-Related Quality of Life Measured after 12 Months

**Figure 13:**
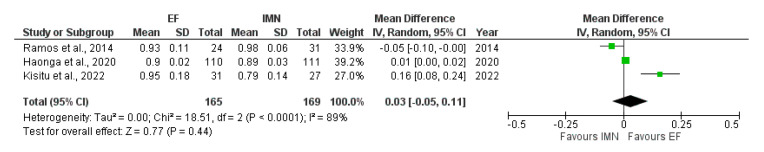
Forest plot presenting the comparison of health-related quality of life measured after 12 months between EF and IMN; Green squares—the individual study effect; Diamond—pooled effect [25,26,27].

Data on health-related quality of life measured after 12 months were provided by three studies [24,27,28] included in the meta-analysis. Due to high heterogeneity (I^2^ = 89%) among included studies, random-effect analysis was performed, and the results showed that no statistical difference was found in the presence of quality of life measured after 12 months between EF and IMN groups (RR = 0.03, 95%CI = −0.05–0.11, *p* = 0.44) (Figure 13).

## 5. Discussion

The findings of this meta-analysis showed that EF was superior in the treatment of open tibia fractures as it was associated with lower rates of hardware failure and pain, but it was inferior in terms of higher rates of superficial infection and malunion, as well as lower health-related quality of life 3 months after treatment. Furthermore, our findings demonstrated no significant difference in deep infection, delayed union and nonunion rates, time to union, and health-related quality of life 12 months following therapy between EF and IMN groups.

The debate over the best surgical treatment technique for open tibia fractures has still been challenging and a subject of discussion for a long time. In 2015. Foote et al. [6] published a network meta-analysis with the objective to compare the relative effects of various treatment options on the risk of requiring unscheduled revision surgery after open fractures of the tibial diaphysis. Independent of the Gustilo classification of the fracture, Foote et al. [6] discovered that the unreamed nail fixation was associated with a lower risk of reoperation in contrast to EF. Results of the meta-analysis conducted by Foote et al. [6] further validated current knowledge that EF of open fractures was associated with increased infection rates [38], many of which may require reoperation. As secondary study endpoints were to quantify differences in malunion, deep infection, and superficial infection in unreamed nail fixation and EF, Foote et al. [6] were unable to identify any significant differences between treatment options due to the small number of reported events. Not long after, in 2016. a research paper reflecting the current status of both IMN and EF for treating Gustilo grade IIIB open tibial fractures was published [15]. This study’s meta-analyses demonstrated similar results to Foote et al. [6], moreover that unreamed IMN was superior in the treatment of Gustilo IIIB tibial fractures, with shorter time to union and decreased incidence of superficial infection and malunion, while without increasing the risk of delayed union, non-union, deep infection, and fixation failure compared to EF.

As meta-analyses of RCTs are regarded to provide the strongest evidence for clinical interventions, as compared to observational studies and solitary randomized trials, it is of immense importance to mention Fu et al.’s [13] 2018. meta-analysis of RCT, aimed to compare EF with tibial nailing treatment modality of open tibial fractures. Six RCTs with a total of 407 cases were included in the Fu et al.’s [13] meta-analysis and their results suggest that nailing treatment is superior to EF in preventing post-fixation complications, such as superficial infection and malunion. Consistent with previously published research, their meta-analysis revealed a higher incidence of superficial infections among EF patients, however, Fu et al. [13] pointed out that with effective debridement, stringent wound management, and prudent antibiotic use the risk of superficial infections among these patients can be reduced to a manageable level. In contrast to superficial infection, deep infections and osteomyelitis are more concerning complications of open tibia fractures treatment. Due to exposure of the marrow cavity, the risk of its further contamination and even amputation is increased in patients treated by IMN. However, in EF patients, osteomyelitis is more often presented after early infection and massive soft-tissue defects and is rarely developed from the pin tract infection. Antibiotic-coated nails have recently been introduced to address this issue since they deliver a high concentration of local antibiotic elution in addition to providing stability at the fracture site. A recent study carried out by Rohilla and colleagues [26] found that both the antibiotic-coated tibial interlocking nail and ring fixator obtained equivalent rates of union and similar complications. The findings of this study showed that, although ring fixation is a well-established alternative for treating open tibial fractures, an antibiotic-coated intramedullary nail may also be a viable choice.

EF patients, on the other hand, had a much lower incidence of hardware failure compared to patients in whom nailing was the treatment modality. With a frequency of up to 3–16%, hardware failure remains the most often reported nailing complication across studies, due to the breakage of locking screws [13]. A total of 22 cases of nailing hardware failure were reported in the study by Fu et al. [13], compared to the six EF cases. Additionally, results of the meta-analysis conducted by Fu et al. [13] showed similar treatment effects for postoperative deep infection, delayed union, and nonunion between groups. The pooled results of the most recent meta-analysis including 9 RCTs with 733 cases [16], suggested that the IMN technique had a lower postoperative superficial infection and malunion rate (RR = 2.84, 95%CI = 1.83–4.39 and RR = 3.05, 95%CI = 2.06–4.52, respectively), but higher hardware failure occurrence in contrast to EF (RR = 0.38; 95%CI = 0.17–0.83). No significant differences were found in the union time, delayed union or nonunion rate, and postoperative deep infection rate between the treatments. These findings are in accordance with the results of our meta-analyses based on 16 RCTs and 1011 cases, where patients treated with IMN had higher rates of hardware failure, however lower rates of complications such as superficial infection and malunion. It is worth mentioning that although several RCTs compared health-related quality of life and pain between EF and IMN patients, no meta-analysis has been performed in this regard. In 2014, a randomized prospective study comparing the Ilizarov circular fixator and locked IMN methods in patients with tibia shaft fractures was published [28]. Results of Ramos et al. [28] demonstrated that despite the fact that the Ilizarov group had more open fractures, the absolute number of severe complications was greater in the IMN group. With respect to assessing major complications between groups, such as compartment syndrome, deep infection, hardware failure, delayed union, pseudarthrosis, and malunion, several self-report measures of pain and functionality were included in this study additionally. There was a statistically significant difference between the Ilizarov and IMN groups of patients on both pain and satisfaction at 1 year, showing that patients in the Ilizarov group scored better than patients in the IMN group. Ramos et al. [28] concluded that diaphyseal tibial fractures may be effectively treated utilizing the Ilizarov method, a minimally invasive procedure that allows for immediate weight-bearing, tend to decrease anterior knee discomfort and benefit patients with leaving no implant behind.

In addition to assessing the composite primary event of reoperation or mortality for deep infection, nonunion, or malalignment at 1 year, Haonga et al. [24] assessed the quality of life between uniplanar EF and IMN patients. The results of this RCT conducted at a tertiary orthopedic center in Tanzania showed significant early differences in quality of life in favor of IMN, however, these differences did not persist at 12 months. Haonga et al. [24] stated that given the inconveniences of an external fixator, the improved early quality of life following intramedullary nailing is not unexpected. Despite variations in radiographic healing and ultimate alignment, quality of life in the treatment groups equilibrated between 6 and 12 weeks, the time during which external fixators were removed. Our meta-analysis, to our knowledge, is the first to evaluate both pain and health-related quality of life, as important outcome measures after open tibia fracture treatment. Pooled results of our meta-analysis revealed a significant difference in quality of life 3 months after the procedure between the EF and IMN groups, favoring IMN. However, the assessment of quality of life evaluated 12 months following interventions presented findings comparable to Haonga et al. [24]. Given that only four studies assessing pain and three studies assessing health-related quality of life were eligible for meta-analysis, more high-quality RCTs should be conducted to provide reliable evidence-based data for clinical decision-making.

There are several limitations of this meta-analysis. The first limitation of this study was that out of the 16 studies included, 7 studies showed a high overall risk of bias. Next, six studies had the method of randomization based on even or odd medical record numbers, not meeting the strict randomization criteria. Furthermore, only 3 studies provided data of pain and health-related quality of life, therefore more RCTs are needed to generate more convincing evidence.

## 6. Conclusions

This meta-analysis presented reduced incidence rates of superficial infection, malunion, and health-related quality of life 3 months after treatment in IMN. However, EF led to a significant reduction in pain and incidence rate of hardware failure. Postoperative deep infection, delayed union, nonunion and health-related quality of life 12 months following therapy were similar between groups. More high-quality RCTs should be conducted to provide reliable evidence-based data for clinical decision-making.

## Figures and Tables

**Table 1:** Characteristics of studies included in the meta-analysis.

Study	Year	Country	No. of Patients		Gender (Male/Female)		Age (Yrs), Mean ± Sd		Follow Up (Month), Mean ± Sd		GA	Type	
			IMN	EF	IMN	EF	IMN	EF	IMN	EF		IMN	EF
Holbrook et al. [25]	1989	USA	29	28	NA	NA	28 (15–66) ꭝ	25 (7–65) ꭝ	16.8 (14–21) ꭝ	18.5 (12–24) ꭝ	I, II, III	Ender	Half-pin
Rohilla et al. [26]	2022	India	16	16	13/3	13/3	33.1 ± 11.2	31.1 ± 9.7	24.1	23.3	II, III	Antibiotic, interlocking	Standard ring frame
Kisitu et al. [27]	2022	Uganda	31	24	21/10	16/8	39 ± 11	39 ± 13	12	4.5	II, IIIa	Unreamed	NA
Haonga et al. [24]	2020	Tanzania	111	110	98/13	91/19	33.3 ± 11.8	31.8 ± 9.5	12	12	I, II, IIIa	Hand-reamed, interlocking (SIGN)	AO uniplanar DISPOFIX
Ramos et al. [28]	2014	Sweden	27	31	19/8	22/9	38 (19–70) ꭝ	46 (18–71) ꭝ	12	12	I, II;	Reamed, locked. cannulated (Syntes)	Original Ilizarov design
Inan et al. [8]	2007	Turkey	29	32	24/5	28/4	31.7 (17–54) ꭝ	32.3 (15–64) ꭝ	43.3 (30–61) ꭝ	46.5 (33–67) ꭝ	IIIa	Unreamed	Hybrid Ilizarov
Li Y et al. [29]	2014	China	46	45	41/5	37/8	44 (18–78) ꭝ	43 (20–82) ꭝ	14.6 (13–17) ꭝ	14.6 (12–17) ꭝ	I, II	Reamed and static locking	Combined with limited open reduction and absorbable internal fixation
Garg et al. [30]	2019	India	25	25	18/7	19/6	Mean: 40.4	Mean: 38.8	36 weeks *		IIIa, IIIb	Unreamed	Half-pin
Mohseni et al. [31]	2011	Iran	25	25	20/5	22/3	30.8 ± 5.2	28.9 ± 8.9	12	12	IIIa, IIIb	Unreamed	AO tubular external fixation
Braten et al. [32]	2005	Norway	36	39	NA	NA	43 (16–90) ꭝ	41 (16–83) ꭝ			I, II	Grosse-Kempf reamed	Ex-fi-re device
Henley et al. [33]	1998	USA	104	70	79/21	53/15	33 (14–81) ꭝ	33 (16–77) ꭝ	472 days	529 days	II, IIIa, IIIb	Unreamed interlocking	Half-pin
Tu et al. [34]	1995	Taiwan	18	18	30/6 *		38.5 (16–65) *ꭝ		20.5 (18–24) *		IIIa, IIIb	Unreamed interlocking	Hoffmann skeletal fixation
Tornetta et al. [23]	1994	USA	15	14	11/4	9/5	41 (21–73) ꭝ	37 (19–86) ꭝ	21 (19–36) *ꭝ		IIIb	Non reamed, statically locked (Gross-Kempf, Alta, AO)	Hoffmann anterior and ACE multiplane
Rodrigues et al. [35]	2014	Brazil	26	31	24/2	28/3	30.5 ± 2	30.3 ± 2.2	12	12	I, II, IIIa	Reamed	Biplanar
Frihagen et al. [36]	2020	Norway	32	31	22/10	20/11	41.8 ± 14.7	43.4 ± 13.5	24	24	42 A-B ﮺	Reamed, locked	TSF ring
Esan et al. [37]	2014	Nigeria	20	20	17/3	16/4	38.1 ± 16.3	40.7 ± 17.1	24	24	II, IIIa	Interlocking (SIGN)	AO/ASIF and Orthofix

NA, Not available; GA—Gustilo-Anderson classification; SIGN—The Surgical Implant Generation Network; AO/ASIF—Association for Osteosynthesis/Association for the Study of Internal Fixation; TSF—Taylor Spatial Fixator. * information for all patients included in study, not according to treatment. ꭝ median (range) ﮺ AO/OTA classification.

## Data Availability

The data that support the findings of this study are available on request from the corresponding author.

## Supplementary Materials

The following supporting information can be downloaded at: https://www.mdpi.com/article/10.3390/medicina59071301/s1, Table S1: Prisma Checklist [39,40]; Table S2: AMSTAR 2 Checklist; Figure S1: Funnel plots of: (**a**) Superficial infection; (**b**) Deep infection; (**c**) Delayed union; (**d**) Malunion; (**e**) Nonunion; (**f**) Hardware failure; (**g**) Pain.
