# Supplementary material for: Updated Meta-Analysis of Randomized Controlled Trials Comparing External Fixation to Intramedullary Nailing in the Treatment of Open Tibial Fractures
Source: Medicina (Kaunas). 2023 Jul 14;59(7):1301. doi: 10.3390/medicina59071301 (PMC10383038; doi:10.3390/medicina59071301)
Supplement: Supplementary file 1 [file medicina-59-01301-s001.zip › Supplement Material Figure S1.pdf]

SUPPLEMENT MATERIAL 1

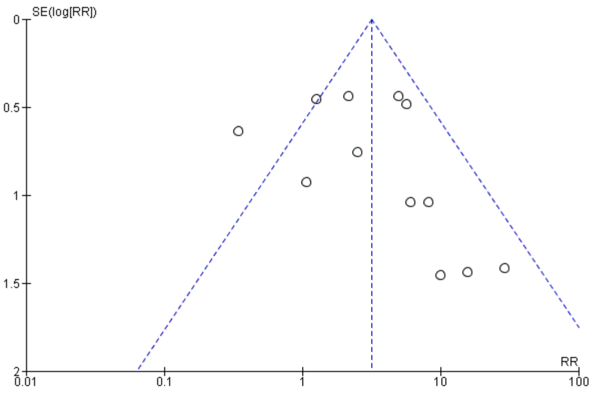

(a)

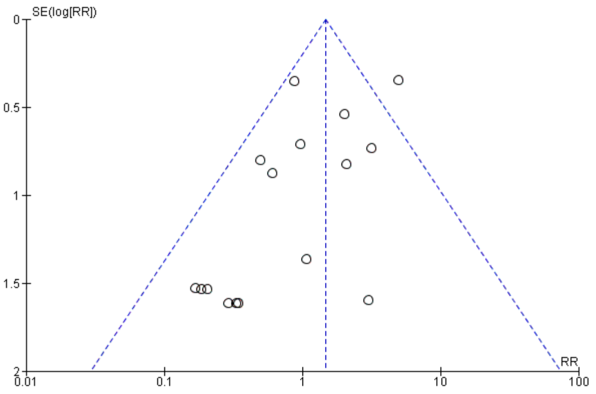

(b)

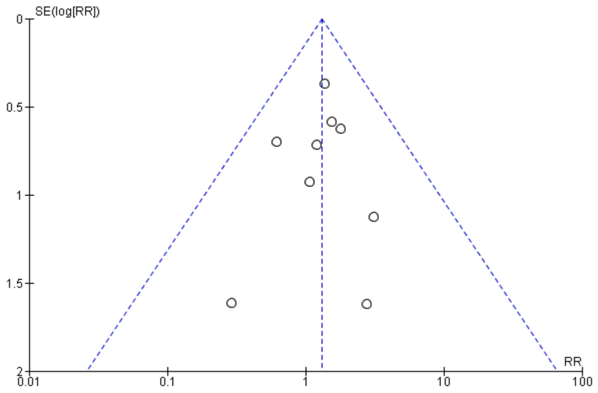

(b)

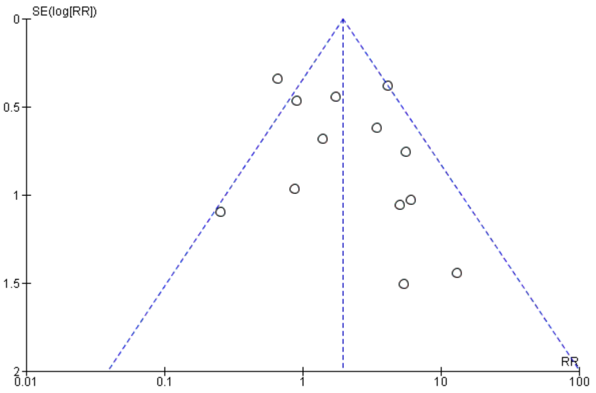

(d)

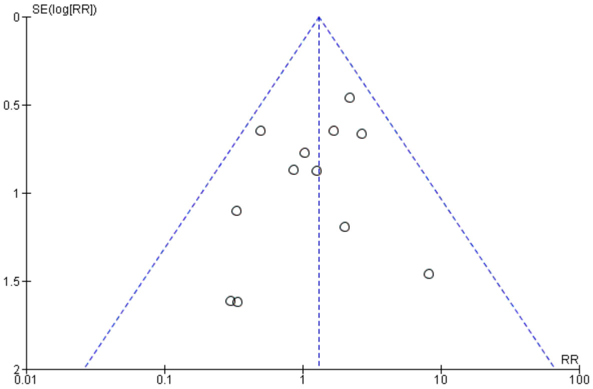

(e)

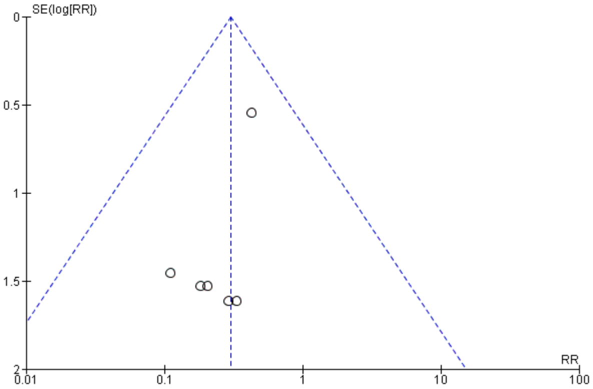

(f)

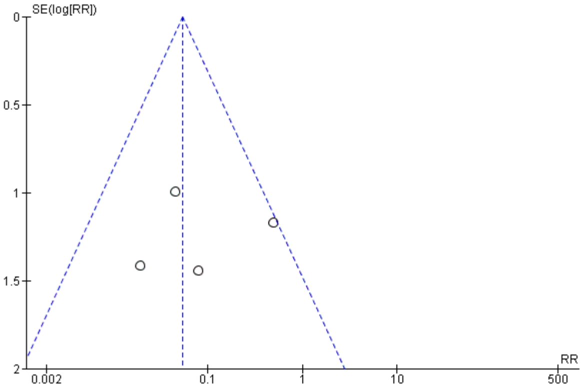

(g)

Figure S1. Funnel plots of: (a) Superficial infection; (b) Deep infection; (c) Delayed union; (d) Malunion; (e) Nonunion; (f) Hardware failure; (g) Pain
